# Growth parameter acquisition and geometric point cloud completion of lettuce

**DOI:** 10.3389/fpls.2022.947690

**Published:** 2022-09-29

**Authors:** Mingzhao Lou, Jinke Lu, Le Wang, Huanyu Jiang, Mingchuan Zhou

**Affiliations:** ^1^College of Biosystems Engineering and Food Science, Zhejiang University, Hangzhou, China; ^2^Key Laboratory of Intelligent Equipment and Robotics for Agriculture of Zhejiang Province, Zhejiang University, Hangzhou, China

**Keywords:** plant factory, plant phenotype, plant growth measurement, 3D reconstruction, point cloud completion

## Abstract

The plant factory is a form of controlled environment agriculture (CEA) which is offers a promising solution to the problem of food security worldwide. Plant growth parameters need to be acquired for process control and yield estimation in plant factories. In this paper, we propose a fast and non-destructive framework for extracting growth parameters. Firstly, ToF camera (Microsoft Kinect V2) is used to obtain the point cloud from the top view, and then the lettuce point cloud is separated. According to the growth characteristics of lettuce, a geometric method is proposed to complete the incomplete lettuce point cloud. The treated point cloud has a high linear correlation with the actual plant height (*R*^2^ = 0.961), leaf area (*R*^2^ = 0.964), and fresh weight (*R*^2^ = 0.911) with a significant improvement compared to untreated point cloud. The result suggests our proposed point cloud completion method have has the potential to tackle the problem of obtaining the plant growth parameters from a single 3D view with occlusion.

## 1. Introduction

With the acceleration of urbanization, the urban population is soaring while the world's arable land has not increased. Food security faces huge challenges with the prediction of the global population predicted to reach 9.8 billion, which means need 60% more food would be needed to feed the world. In the context of the COVID-19 pandemic, novel coronavirus also occurs on produce that is exposed to farmers infected with the virus. An efficient automated factory method of agricultural production is needed to meet global food security demands in the face of uncertainty, such as the COVID-19 pandemic and a growing population (Saad et al., 2021).

The plant factory is a form of controlled environment agriculture (CEA) (R Shamshiri et al., 2018) in the production process of leafy vegetables which can precisely control factors such as the light, water, fertilizer, and carbon dioxide concentration, etc. (Ting et al., 2016). To achieve precise process management and yield prediction in plant factories, plant growth parameters such as plant height, leaf area, and fresh weight need to be measured accurately and non-destructively in real time. These plant growth parameters provide effective feedback for precise control of the plant factory. Manual measurements are laborious and time-consuming;, therefore new automatic measurements are required (Li et al., 2020).

There are many ways to extract the growth parameters of plants, such as methods based on 2D image techniques and methods based on 3D vision. 2D image, including RGB image and hyperspectral- based phenotyping, offers several advantages (Campbell et al., 2015): (1) Quantitative measurements can be recorded over discrete time points and various types of spectral imaging provide parameters that the human eye cannot capture; and (2) For the detection and monitoring of plant disease, an imaging system combined with hyperspectral technology is a good approach (Mahlein et al., 2012). Researchers acquired 2D images with simple and inexpensive equipment, using pixel counts to estimate plant growth parameters (Easlon and Bloom, 2014; González-Esquiva et al., 2017; Tech et al., 2018; Pérez-Rodríguez and Gómez-García, 2019; Nasution et al., 2021). The fresh weight of plants can also be estimated by image processing technology and function model. Jiang et al. (2018) proposed a real-time image processing and spatial mapping method to estimate the fresh weights of lettuce individual. The plant weight was estimated by calibration equations that relate the pixel numbers of lettuce images to their actual fresh weights with a two-point normalization method. Ge et al. (2016) processed RGB images to estimate projected plant area, which are correlated with destructively measured plant shoot fresh weight, dry weight, and leaf area. Campbell et al. (2020) modeled shoot growth as a function of time and soil water content by calculating the projected shoot area from the RGB images. Bai et al. (2016) post- processed RGB images to extract canopy green pixel fraction as a proxy for biomass. In conclusion, 2D image method has advantages of a simple acquisition system, requiring less computation resources, which can provide the required results in real time. However, it cannot handle crops with complex structures, and the variety of growth information that can be obtained is limited.

3D vision methods can be used for estimating plant phenotypic parameters. Building on previous research (Yeh et al., 2014), Chen et al. (2016) designed and developed an automated measurement system, includinge a weight measurement device and an imaging system, which proved that stereovision can be used to estimate plant weight. But the proposed method was still unable to solve the occlusion problem between the blades, and the errors increased with growingover time. To solve the problem of occlusion, Blok et al. (2021) estimated the size of field-grown broccoli heads based on RGB-Depth (RGB-D) images and applied the Occlusion Region-based Convolutional Neural Network (CNN) (Follmann et al., 2019). This method could predict the size of broccoli for different varieties under a high degree of occlusion, but the shape of broccoli itself is relatively regular, and the measured size was limited to the diameter. Rose (Rose et al., 2015) reconstructed tomato plants using the Structure from Motion (SfM) and Multi-view Stereo (MVS) methods. The point cloud obtained was highly correlated with the point cloud obtained by the high-accuracy close-up laser scanner. The multi-view method partly solved the problem of occlusion, but it is very time-consuming to calculate point clouds. In conclusion, 3D vision methods have certain advantages in measuring plant phenotypic parameters, but there are still inevitable occlusion problems, and some existing multi-perspective methods are time-consuming.

Deep learning has been widely used in the domain of digital image processing to solve difficult problems. In the field of 3D shape completion, deep learning was initially applied to 3d objects represented by voxels (Wu et al., 2015; Li et al., 2016; Sharma et al., 2016; Dai et al., 2017; Han et al., 2017). Since point clouds are more portable than voxels, many scholars have studied how to apply deep learning networks to point clouds in recent years, such as AtlasNet (Vakalopoulou et al., 2018), PCN (Yuan et al., 2018), and FoldingNet (Yang et al., 2018). The benefit of deep learning is that it can achieve the point cloud completion while reducing the progress of geometrical modeling. However, the drawback is that a big amount of data is required to get a well-trained network, meanwhile, the different type of plant, contributes to an even more complex data set preparation and network tuning overhead. Furthermore, the ground truth of the plant point cloud is very hard to obtain even with multi view 3D vision due to the complicated occlusion.

This paper addressed the problem to obtainof obtaining the growth parameters of a plant from a single 3D view with occlusion. Taken Taking the physical and growth characteristics of lettuce as an example, the focus of our research is to complete the point cloud with a geometric method in a single 3D view. Then the completed point cloud is used to estimated the growth parameters with regression. The contributions of this paper are listed as followspaper is organized as follows,

- A framework is proposed for real-time nondestructive detection of plant growth parameters in a plant factory.
- A point cloud completion method is proposed based on a single 3D view. The regression of the plant growth parameters is obtained with the completed point cloud instead of using an original single view point cloud.
- The experimental results show the accuracy and feasibility of the proposed method in the plant factory.

The remainder of the paper is organized as follows: The proposed method and experimental setup are described in Section 2. In Section 3, the performance of the proposed method is evaluated. Finally, Section 4 discusses the advantages and disadvantages of the proposed method and presents future work.

## 2. Materials and methods

### 2.1. The work flow of the proposed method

The raw plant point cloud was obatinedobtained from the top view through ToF camera. Preprocessing and segmentation are carried out first. Through pass-through filtering, the region of interest was concentrated in the plant location according to the XYZ coordinate value of the point cloud. And the region of interest (ROI) was clustered using color-based region growing segmentation method to obtain the plant point cloud and soil point cloud respectively. Then the statistical analysis method was used to filter out the noise points by: calculatinge the distance distribution between the point and all the points in the domain, and deletinge the points whose distance was greater than the standard deviation threshold.

**Figure 1:**
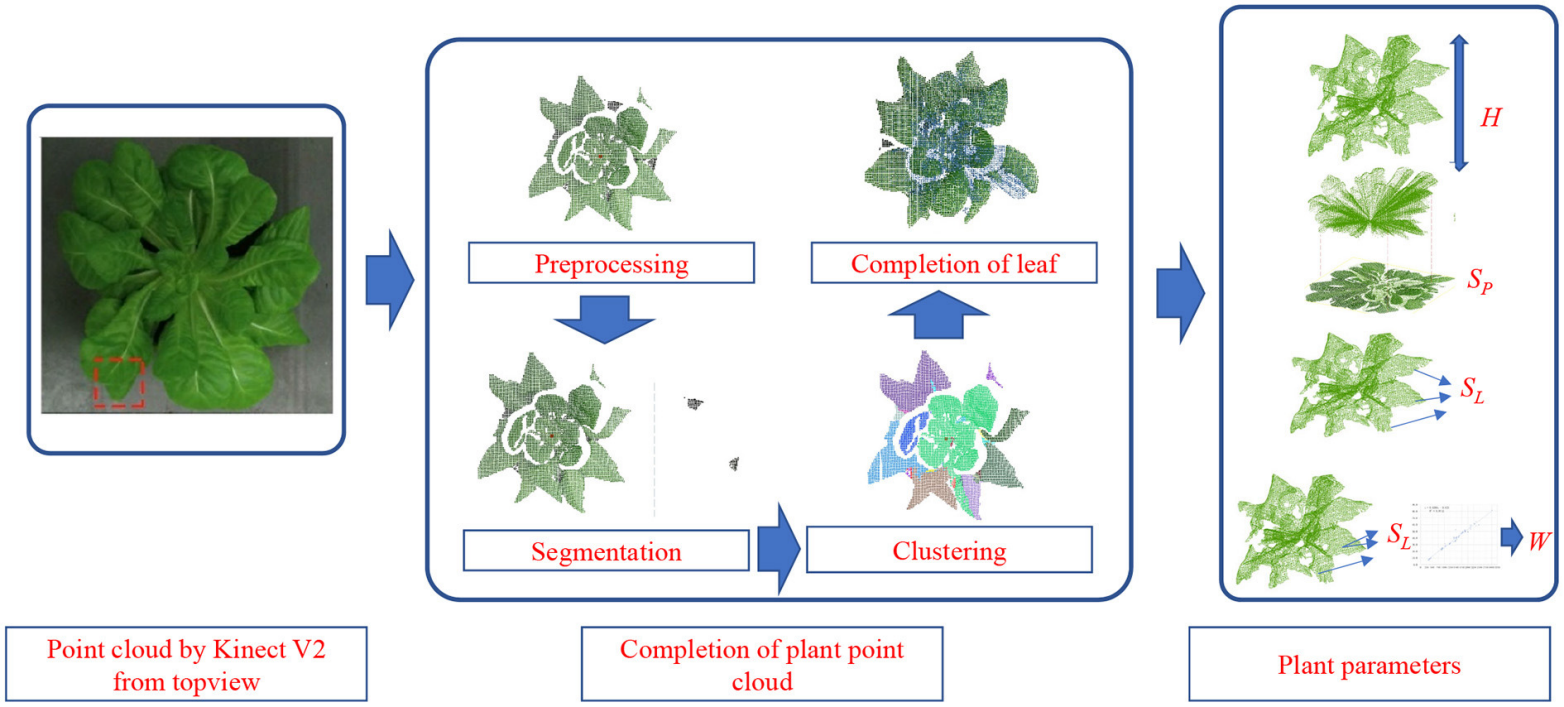
The workflow of the completion of plant point cloud and the acquisition of plant growth parameters.

Afterwards, the completion of the point cloud was carried out considering the kinds of symmetric leaf and asymmetrical leaf. With the completed point cloud, the plant parameters, including plant height, project area, total leaf area, and fresh weight, are estimated with regression. The overall workflow of the proposed method is shown in Figure 1. After completing the point cloud, considering the possible problem of repeated sampling, the downsampling of the completed point cloud was carried out according to the set sampling radius.

### 2.2. Plant point cloud acquisition

**Figure 2:**
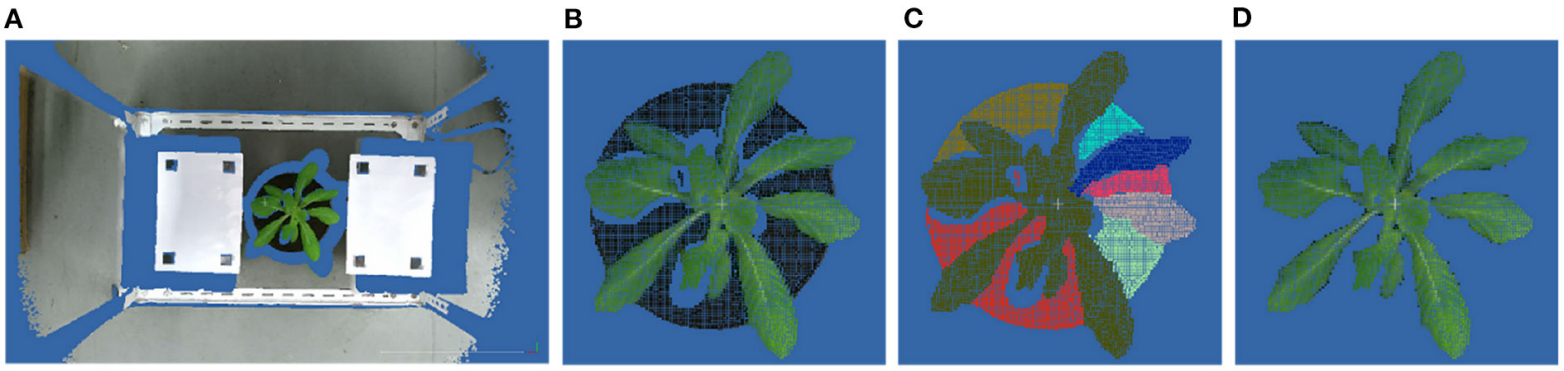
Point cloud preprocessing results. **(A)** The original point cloud obtained by ToF camera from the top view. **(B)** ROI point cloud obtained by pass- through filter. **(C)** Plant and soil point clouds obtained by color-based region growing segmentation. **(D)** Plant point cloud after segmentation.

Under experimental setting conditions, the location of the shooting area relative to the camera was fixed. So, the pass- through filter can be used to get the point cloud of the region of interest (ROI) from the original point cloud. The point cloud of the ROI mainly includes plant point cloud and soil point cloud:

(1) $$\begin{array}{l} \begin{array}{c} P_{ROI}=P_{plant}+P_{soil}, \end{array} \end{array}$$

where *P*_*ROI*_ is the set of points in ROI, and *P*_*plant*_ and *P*_*soil*_ are the sets of points of plant and soil, respectively. Because of the color difference, the color-based region growing segmentation method can be used to get point clouds of the soil and the plant. Plant point cloud acquisition workflow is shown in Figure 2.

### 2.3. Plant point cloud completion

Since lettuce leaves groew outward from the center of the root and the ToF camera was located above the lettuce, the obtained point cloud has a deletion near the root caused by occlusion. The core of the completion method is to complete the holes between the root and the known leaf point cloud. However, since the position of the root cannot be directly obtained from the camera's perspective, it needs to be estimated. The position of the root point in space (*p*_*root*_) can be represented by three-dimensional coordinates, that is, *p*_*root*_ can be represented by {*x, y, z*} in the camera coordinate system. The X-axis and Y-axis are parallel to the X-axis and Y-axis of the camera's imaging plane, respectively, and the Z-axis is perpendicular to the imaging plane.

Because of the flat soil surface, the height of the root point can be determined by the height of the soil plane. Due to the uniform lighting in the plant factory, we can consider that lettuce leaves grow evenly outward. Therefore, the specific position of the root point in the soil plane can be obtained by calculating the centroid of the lettuce from the top view. The equations are as follows:

(2) $$\begin{array}{l} \;p_{root}=\left\{x,y,z\right\}\\ x=x_{c},y=y_{c},z=\frac{\sum_{p\in P_{soil}}z_{p}}{\left|P_{soil}\right|}, \end{array}$$

where *x*_*c*_ and *y*_*c*_ are the *x* value and *y* value of plant centroid respectively. *P*_*soil*_ represents the soil point cloud.

After the root location coordinates are obtained, the existing plant point clouds cannot be directly used for completion. It is necessary to separate the single leaf point cloud (*P*_*leaf*_) from the plant point cloud first. The accuracy of point cloud of separated leaves will directly affect the accuracy of leaf area and weight prediction after completion. Color-based region growing segmentation method can also be used for single leaf segmentation. Reduce the distance threshold and color threshold to segment every leaf point cloud.

**Figure 3:**
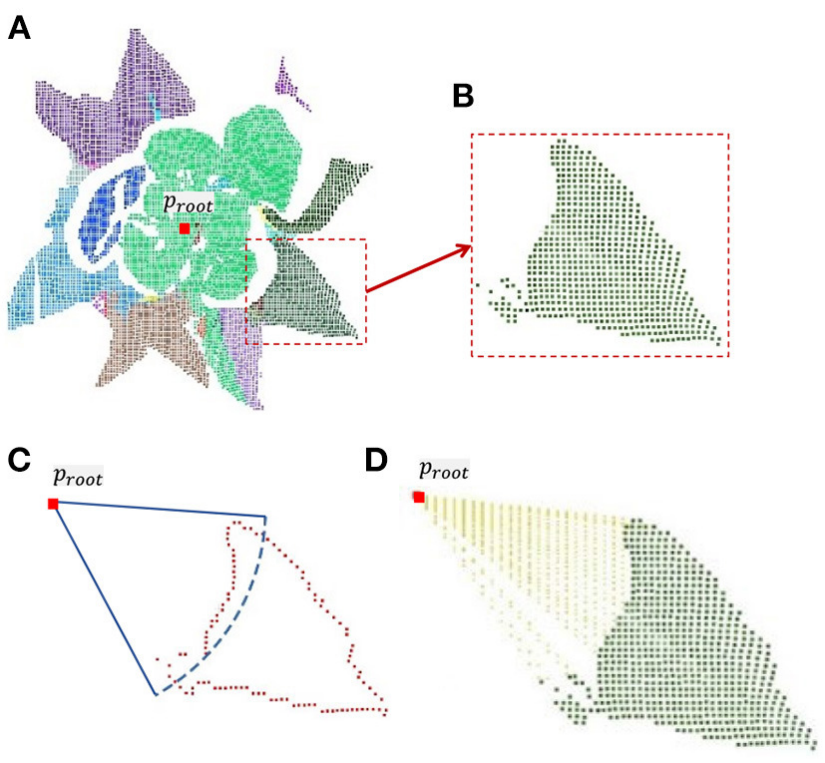
Completion of a single leaf point cloud. **(A)** Leaf point clouds were obtained from plant point cloud by color-based region growing segmentation. **(B)** Symmetrical leaf point cloud. **(C)** According to the calculated threshold, the edge points in the leaf point cloud are determined. **(D)** Result of uniform sampling between edge points and root point.

Lettuce leaves grow from the inside out, so the central part of the leaves are is less sheltered. If all the leaves were completed, the estimated leaf area would be larger than the true value. Therefore, only the outer leaves will be completed. Calculate the distance between the centroid of every single leaf (*P*_*leaf*_) and the root point (*p*_*root*_). Only when the distance is greater than the threshold, it isis it considered that the leaf is far from the center, which has serious occlusion and needs to be completed. The key steps of plant point cloud completion are shown in Figure 3.

#### 2.3.1. Symmetric leaf point cloud completion

**Figure 4:**
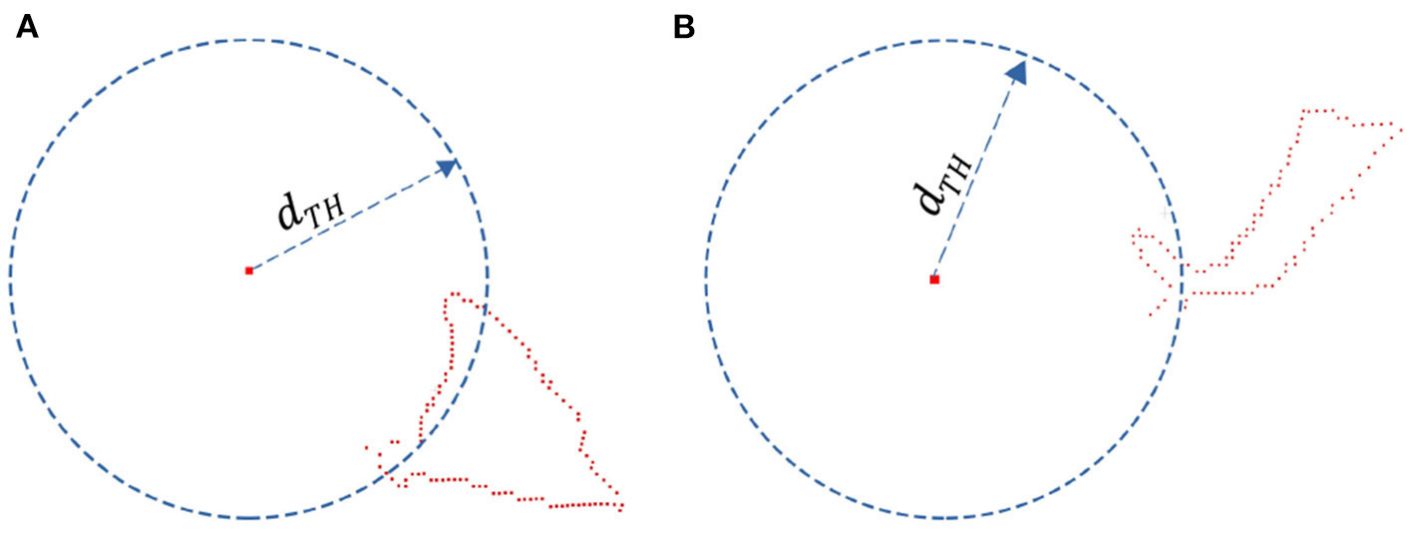
The determinate closest edge. **(A)** Symmetrical leaf. **(B)** Asymmetrical leaf.

The core method of leaf point cloud completion is to find the closest edge of the point cloud to the root point and then add missing points between the edge and the root point by uniform sampling. The first step is to determine the closest edge by distance threshold segmentation. The distance threshold can be determined by the following equation,

(3) $$\begin{array}{l} \begin{array}{l} d_{TH}=d_{min}+var\text{\_}mul*\sigma \\ d_{min}=|p,p_{root}{|}_{min},p\in P_{leaf}\\ \;\sigma =\sqrt{\frac{\sum_{p\in P_{leaf}}|p,p_{root}|}{|P_{leaf}|-1}} \end{array} \end{array}$$

where |*p, p*_*root*_| is the distance from point in single leaf point cloud *P*_*leaf*_ to the root point *p*_*root*_. *d*_*min*_ is the minimum distance and σ is the standard deviation. *var*_*mul* is an artificially defined, adjustable parameter. The larger the parameter, the more points on the closest edge. Segmentation results of the closest edge points are shown in Figure 4. In order to solve the problem of repeated sampling caused by edge points on the same sampling line, edge points need to be screened.

The leaf tip may be asymmetrical due to the variety of occlusion conditions. If the segmented leaf tip is asymmetric, there will be a big gap between the completed point cloud and the original point cloud, shown in Figure 4. The completion method of asymmetric leaves will be explained in the following section.

#### 2.3.2. Asymmetric leaf point cloud completion

**Figure 5:**
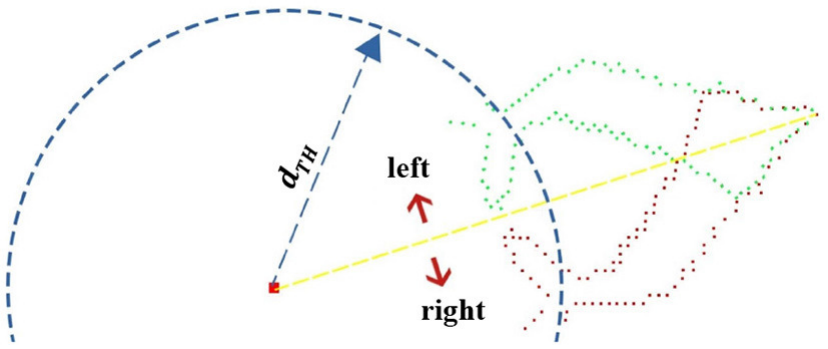
Completion of the asymmetric leaf.

**Figure 6:**
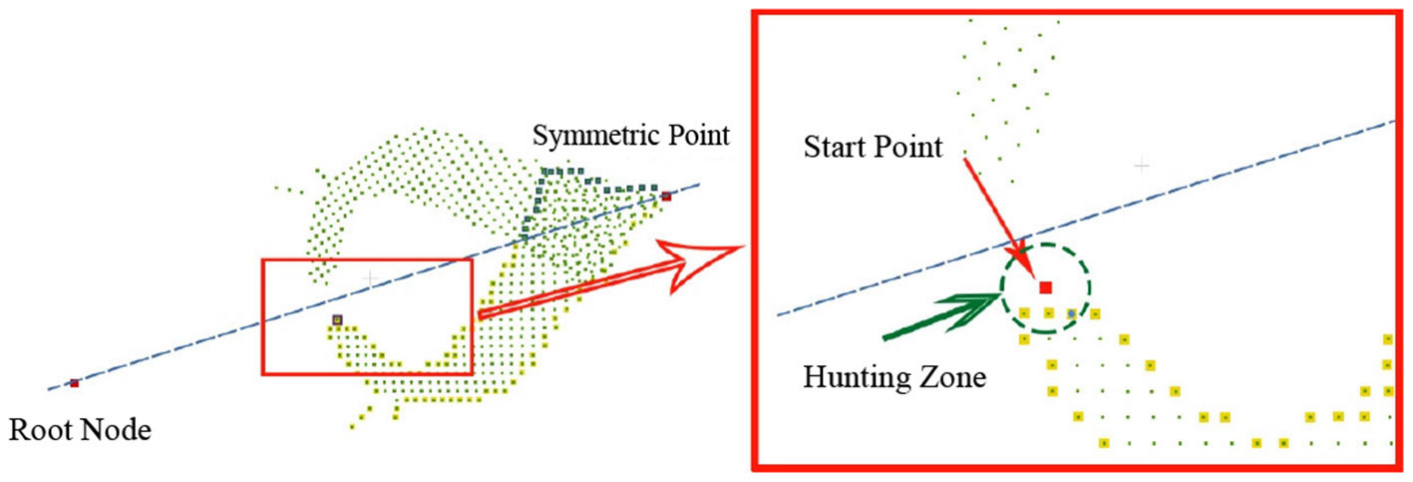
Search the hole boundary of asymmetrical leaf.

For leaves severely occluded on one side, the occluded part can be restored from the point cloud features of leaves on the other side by mirroring. The first step in mirroring is to determine the axis of symmetry. The axis of symmetry can be approximated as the line between the farthest point in single leaf point cloud (*P*_*leaf*_) and root point *p*_*root*_, for example, the yellow line in Figure 5. The symmetry of the original leaf point cloud depends on the following inequality,

(4) $$\begin{array}{l} \begin{array}{c} \frac{2\times ||P_{left}|-|P_{right}||}{\left|P_{left}|+|P_{right}\right|}<\mu , \end{array} \end{array}$$

where |*P*_*left*_| is the number of points on the left side of the line, and |*P*_*right*_| is the number on the right side. μ is a set threshold (we set it as 0.23). When the judgment condition is false, the leaf is considered to be asymmetric. Only the point cloud at one side of the symmetry axis with more points is selected for transformation. The mirror transform result is shown in Figure 6. Obviously, there may still be holes in the leaf point cloud after transformation. So, the leaf needs to be checked for holes and filled.

The proposed method is to find the edge points of the hole in the point cloud at one side of the symmetry axis, and then sample evenly between these edge points and their mirror points to obtain the completed point cloud. The flow to find edge points is as follows:

(1) Determine the start point: All points in the point cloud at one side of the axis of symmetry are sorted according to the relative distance of the root point to obtain an ordered point set with the number of *n*_*s*_ (we set it as 20) points closest to the root point (*P*_*O*_). In *P*_*O*_, the point closest to the axis of symmetry is selected as the start point (*p*_0_). Put *p*_0_ in the set of boundary points (*P*_*edge*_).

(2) Search all edge points: *p*_0_ is considered as the first search point. All points in the same side point cloud with radius *r* near the current search point are searched through K-dimensional tree to obtain the adjacent point set (*P*_*ad*_). Calculate all points in *P*_*ad*_ are calculated and delete points whose distance is less than the current search point according to the distance from root point are deleted. From the remaining points, the closest point to the axis of symmetry is selected as the next edge point. Put it in *P*_*edge*_ and make this point is made as the next search point. Repeat this step until the next search point cannot be selected.

**Figure 7:**
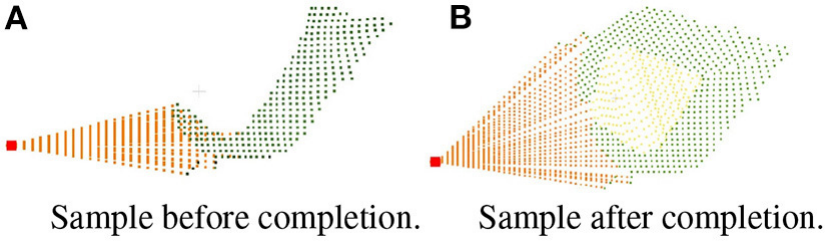
Comparison of effect before and after completion. **(A)** Sample before completion. **(B)** Sample after completion.

The pseudo code of the symmetry judgment and completion of single leaf point cloud is shown in Algorithm 1. Then the leaf is completed the same as the symmetric leaf through finding the closest edge and complete the missing points are completed between the edge and the root point by uniform sampling. To solve the problem of repeated sampling in the process of leaf point cloud completion, a down-sampling is required after the completion of the whole plant. The comparison of effect before and after completion is shown in Figure 7.

**Algorithm 1:** Leaf point cloud completion.

**INPUT:** *P*_*leaf*_ - Leaf point cloud *p*_*f*_ - Leaf tip point *p*_*root*_ - Root point **OUTPUT:** *P*_*complete*_ - completed leaf point cloud
**function** symmetry(*P*_*leaf*_, *p*_*f*_, *p*_*root*_)
2: **for all** *p* such that *p* ∈ *P*_*leaf*_ do
**if** (*y*_*p*_ − *y*_*root*_)(*y*_*f*_ − *y*_*root*_) − (*x*_*p*_ − *x*_*root*_)(*x*_*f*_ − *x*_*root*_) > 0 **then** *P*_*l*_ ← *p* ;
4: **else** *P*_*r*_ ← *p* ;
**end if**
6: **end for**
δ = 2 × (|*P*_*l*_| − |*P*_*r*_|)/(|*P*_*l*_| + |*P*_*r*_|) ;
8: **if** δ > μ **then** *P*_*c*_ = *P*_*l*_ ;
**else if** δ < −μ **then** *P*_*c*_ = *P*_*r*_ ;
10: **else return** *P*_*leaf*_ ;
**end if**
12: *P*_*s*_ ← points obtained by symmetrizing *P*_*c*_ according to the line *p*_*f*_ and *p*_*root*_ ;
*P*_*leaf*_ = *P*_*leaf*_ + *P*_*s*_ ;
14: *P*_*next*_ ← *n*_*s*_ *p* with the shortest distance to *p*_*root*_, *p* ∈ *P*_*c*_ ;
**while** *P*_*next*_ ≠ ∅ do
16: *p*_*current*_ = *p*(*min*(*p, line*(*p*_*root*_, *p*_(*f*)_)), *p* ∈ *P*_*next*_) ;
*P*_*hole*_ ← *p*_*current*_ ;
18: *P*_*next*_ = ∅ ;
**for all** *p* such that |*p, p*_*root*_| > |*p*_*current*_, *p*_*root*_|, |*p, p*_*current*_| < *r*, *p* ∈ *P*_*c*_ **do** *P*_*next*_←*p* ;
20: **end for**
**end while**
22: **for all** *p* such that *p* ∈ *P*_*hole*_ **do** Uniformly sampled ground between *p* and the point of symmetry of *p* ;
**end for**
24: **return** *result*
**end function**

### 2.4. Plant parameters estimation

The following four plant growth parameters need to be estimated, and the estimation method will be presented in sequence: plant height, plant projection area, plant leaf area, and plant weight.

#### 2.4.1. Height estimation

Since the camera was located at the top of the plant, the point cloud at the bottom of the plant cannot be directly obtained, so the estimated height of the plant can be divided into the following two types. Absolute height *H*_*abs*_ is the height from the top of the plant point cloud to the soil point cloud. The relative height *H*_*rel*_ is the height from the top to the bottom of the plant point cloud. The equations are as follows:

(5) $$\begin{array}{l} \begin{array}{l} \text{Absolute height: }H_{abs}=H_{top}-H_{soil}\\ \text{Relative height: }H_{rel}=H_{top}-H_{bottom}, \end{array} \end{array}$$

where *H*_*top*_ and *H*_*bottom*_ are the heights of the highest and the lowest point calculated by comparing the *z* value of all the points. *H*_*soil*_ is the height of the soil obtained by averaging the *z* value of the soil point cloud. *H*_*bottom*_ is the height of the fixed pot edge.

#### 2.4.2. Projected area estimation

The projected area here refers to the area of the completed point cloud projected onto the imaging plane of the camera. For the convenience of calculation, after the projection, the points with uniform distribution in the plane were obtained by down-sampling. The projected area *S*_*pa*_ can be calculated using the following equation:

(6) $$\begin{array}{l} \begin{array}{c} S_{pa}=N_{pa}\times \delta , \end{array} \end{array}$$

Where *N*_*pa*_ is the number of points on the projection plane and, δ is the proportion of area corresponding to a single point.

#### 2.4.3. Total leaf area estimation

Completed point clouds cannot be directly used to estimate plant area parameters. In order to facilitate the estimation, triangulation is required. The method steps are as follows Hu et al. (2018):

1. Generate triangles from the plant point cloud. Triangles need to meet two conditions: Firstly, there are no points in the minimum enclosing ball of each triangle. Secondly, the edges of the triangle are less than the threshold, in order to avoid connecting discontinuous surfaces.
2. Remove abnormal triangles, including redundant paired triangles, suspended triangles, multi-connected triangles, and the triangles nearly perpendicular to surfaces.

After the triangulation, the total area of plant leaves was obtained by calculating the area of all triangles in the triangulated point cloud.

#### 2.4.4. Volume and weight estimation

Since there is a high linear correlation between the volume and weight of lettuce and the total leaf area, it is possible to estimate the weight from the total leaf area.

### 2.5. The ground truth measurement

#### 2.5.1. Height

The values to be measured include absolute height, relative height, projected and total area of the leaves, and weight. The ground truth of absolute height $H_{abs}^{T}$ and relative height $H_{rel}^{T}$ are measured by a steel ruler with accuracy of 1 *mm*. $H_{abs}^{T}$ is the height from the top of the plant to the plane of the pot while the $H_{rel}^{T}$ to the plane of soil.

#### 2.5.2. Projected leaf area

**Figure 8:**
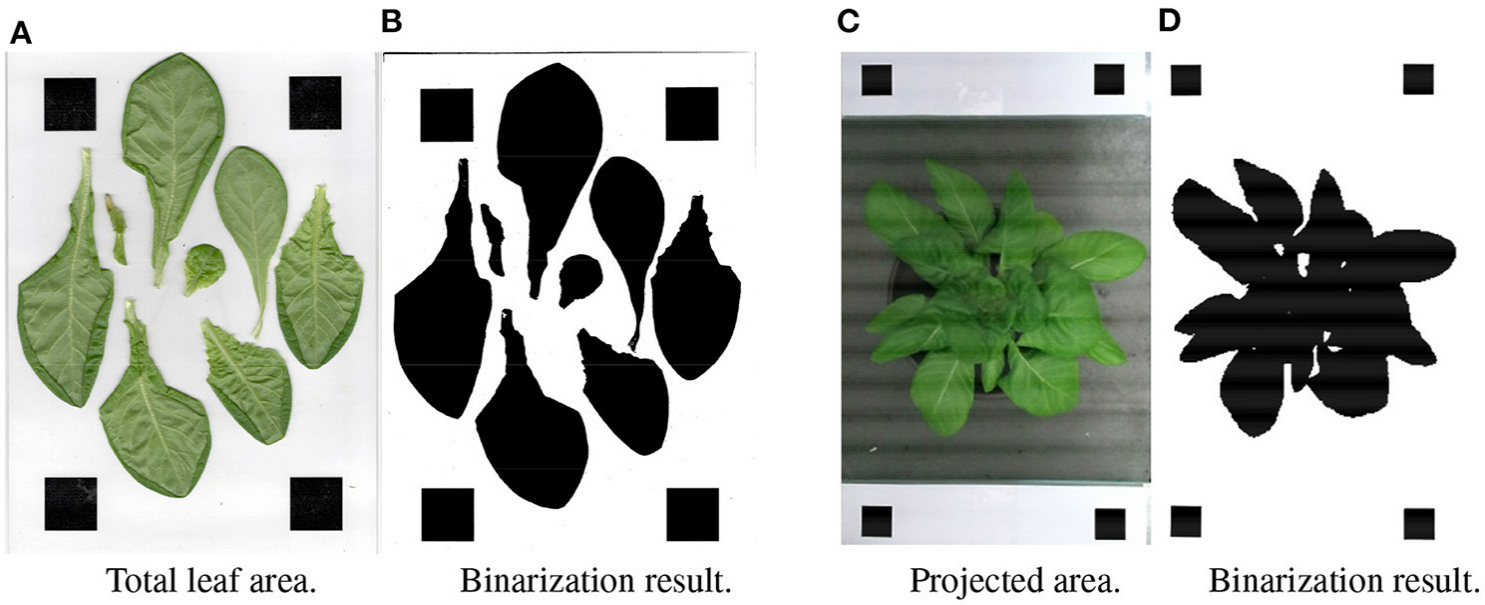
Measurement of the ground truth of the projected area and total leaf area. **(A)** Total leaf area. **(B)** Binarization result. **(C)** Projected area. **(D)** Binarization result.

To measure the projected area of the plant, two pieces of white- paper- printed square markers (the true area is known) were placed on both sides of the pot at the same height. The placement is as shown in Figure 8C. In order to better segment leaves in the image, the RGB image was converted to the excess green model (Søgaard and Olsen, 2003):

(7) $$\begin{array}{l} \begin{array}{c} ExG=2G-R-B, \end{array} \end{array}$$

where *R*, *G*, and *B* are three color channels (red, green, blue, respectively). The *ExG* value of each pixel will be used as the determination criterion for binarization. The binarized result is shown in Figure 8D. And the projected area can be estimated by the following formula:

(8) $$\begin{array}{l} \begin{array}{c} S_{pa}^{T}=\frac{N_{pa}}{N_{m}}\times S_{m}, \end{array} \end{array}$$

where *N*_*pa*_ and *N*_*m*_ are the number of leaves pixel points and markers pixel points in the binary graph, respectively. *S*_*m*_ is the area of the markers.

#### 2.5.3. Total weight and volume

The ground truth weight was obtained by cutting the plant above the soil surface and weighing it with an analytical balance with accuracy of 0.1 *mg*. Considering the irregular shape of the lettuce, the total volume cannot be obtained directly. In this paper, we used the drainage method to measure the lettuce reference total volume. First, we prepared a measuring cylinder with a quantitative amount of water, then put the lettuce into the cylinder to read the change of the water volume in the cylinder, which is the total volume of the lettuce.

#### 2.5.4. Total leaf area

The ground truth of total area was measured by the sum of all the cut leaves. The method is similar to the projected area: all the leaves were tiled on the white paper with markers printed, and the top view photo (Figure 8A) was taken by camera. The result of binarized *ExG* image is shown in Figure 8B. And the leaf area can be estimated by the following formula:

(9) $$\begin{array}{l} \begin{array}{c} S_{a}^{T}=\frac{N_{a}}{N_{m}}*S_{m}, \end{array} \end{array}$$

where *N*_*a*_ and *N*_*m*_ are the number of pixel points of the cut leaves and markers in the binary graph respectively. *S*_*m*_ is the true area of the markers.

### 2.6. Experimental setup

**Figure 9:**
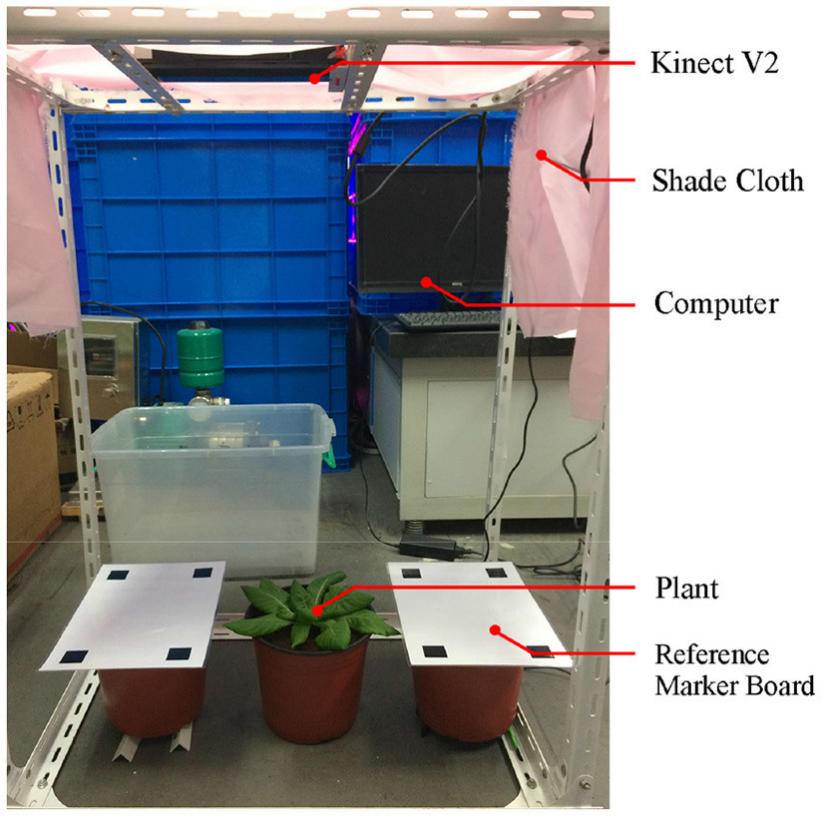
Indoor static plant information collection platform.

The ToF camera was fixed above the experimental setup to obtain point cloud from the top view (shown in Figure 9). The relative height from the plant to ToF camera was 0.6–0.7 *m*. In order to ensure uniform illumination and reduce the influence on the color of plant point clouds from the shadow of the camera, shading cloth was placed on the top and sides of the device. Reference calibration plates with four 20 × 20 *mm* black squares were placed on both sides of the plant. The experimental subjects were 54 lettuces grown in plant factories. The lettuces were divided into six groups of nine plants, with growing periods ranging from 28 to 63 days, 7 days apart.

### 2.7. Hardware devices

The ToF camera used in the experiment is Microsoft Kinect V2 (specifications in Table 1; Fankhauser et al., 2015). The point cloud data obtained by the ToF camera is processed by a laptop computer. The configuration parameters of the computer (MECHROVO X8Ti) are: CPU i7-8500H, GPU GTX 1060, and OS Ubuntu 16.04.

**Table 1:** Kinect v2 specifications.

**Camera**	**Resolution**	**Field of view**	**Operating range**	**Frame rate**
Infrared/depth camera	512 × 424 *px*	70.6° × 60.0°	0.5 − 4.5*m*	30 *Hz*
Color camera	1, 920 × 1, 080 *px*	84.1° × 53.8°		30 *Hz*

### 2.8. Camera calibration

Before using the camera, the checkerboard correction method (Zhang, 2000) was used to correct it. A tripod was used to fix the ToF camera, and another tripod was used to fix the calibration checkerboard. The distance and angle between the camera and checkerboard were adjusted for calibration. From 0.5*m* to 4.5*m*, 100 pictures were taken from the different positions in the field of camera view with the checkerboard arranged at different angles to calculate the internal reference for infrared camera and color camera. The spatial transformation relationship between two camera and the distortion were calculated. The results are shown as follows:

(10) $$\begin{array}{l} \text{Color camera:}\left[\begin{array}{ccc} f_{\alpha } & 0 & u_{0}\\ 20 & f_{\beta } & v_{0}\\ 0 & 0 & 1 \end{array}\right]=\left[\begin{array}{ccc} 1065 & 0 & 947\\ 20 & 1068 & 549\\ 0 & 0 & 1 \end{array}\right] \end{array}$$

(11) $$\begin{array}{l} \text{Depth camera:}\left[\begin{array}{ccc} f_{\alpha } & 0 & u_{0}\\ 20 & f_{\beta } & v_{0}\\ 0 & 0 & 1 \end{array}\right]=\left[\begin{array}{ccc} 362 & 0 & 251\\ 20 & 363 & 208\\ 0 & 0 & 1 \end{array}\right] \end{array}$$

(12) $$\begin{array}{l} \text{Rotation matrix:}R=\\ \left[\begin{array}{ccc} 9.999\times 10^{-1} & -2.901\times 10^{-3} & 2.396\times 10^{-3}\\ 2.396\times 10^{-3} & 2.928\times 10^{-3} & 9.999\times 10^{-1}\\ -2.375\times 10^{-3} & -7.413\times 10^{-3} & 9.999\times 10^{-1} \end{array}\right] \end{array}$$

(13) $$\begin{array}{l} \text{Translation matrix:}t=\\ \left[\begin{array}{c} -5.203\times 10^{-2}-1.823\times 10^{-2}-1.904\times 10^{-1} \end{array}\right] \end{array}$$

Where *f*_α_ and *f*_β_ are the focal length in x and y directions, respectively. *u*_0_ and *v*_0_ are the coordinates of primary point. The primary point is the point where the optical axis intersects the image plane. Rotation matrix and translation matrix guide how to transpose the color map axes to the depth map axes.

## 3. Results

**Figure 10:**
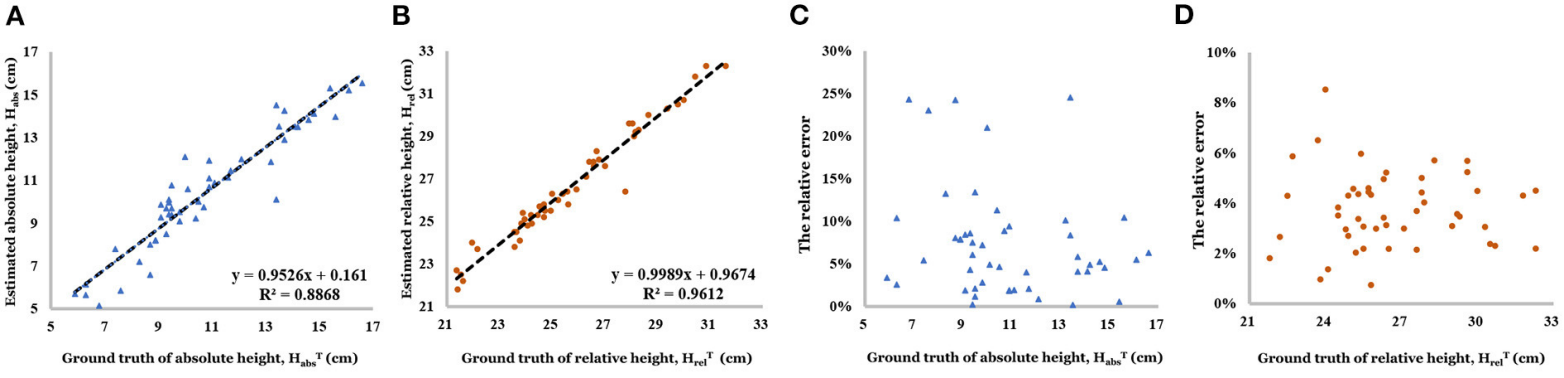
**(A)** Linear relationship between estimated absolute height and ground truth of absolute height. **(B)** Linear relationship between estimated relative height and ground truth of relative height. **(C)** Relative error of absolute height. **(D)** Relative error of relative height.

As shown in Figure 10, linear correlation between the estimated relative height and the actual value (*R*^2^ = 0.961 and 80% of relative error concentrated in the interval of 2.0–6.0%) is better than estimated absolute height (*R*^2^ = 0.887 and 80% of relative error concentrated in the interval of 0–10.0%).

**Figure 11:**
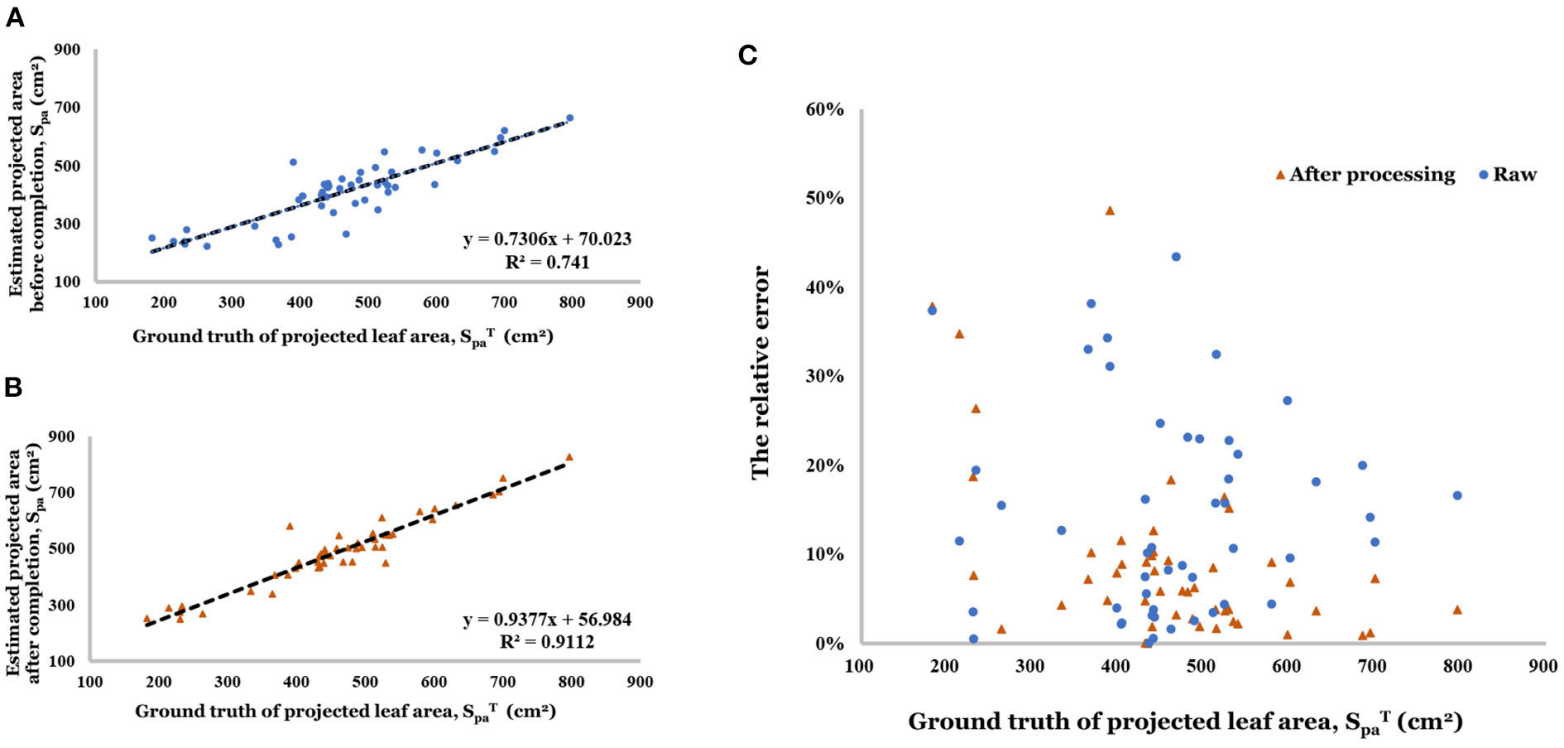
**(A)** Linear regression result of estimated projected area before completion. **(B)** Linear regression result of estimated projected area after completion. **(C)** Relative error of projected area before and after completion.

After completing the point cloud, the estimation accuracy of projected leaf area was greatly improved (*R*^2^ increased from 0.741 to 0.911, in Figures 11A,B). The relative error distribution has been shown in Figure 11C. The range of relative became more concentrated (0% - 9.8%) because of the point cloud completion (0.1–20.0% before).

**Figure 12:**
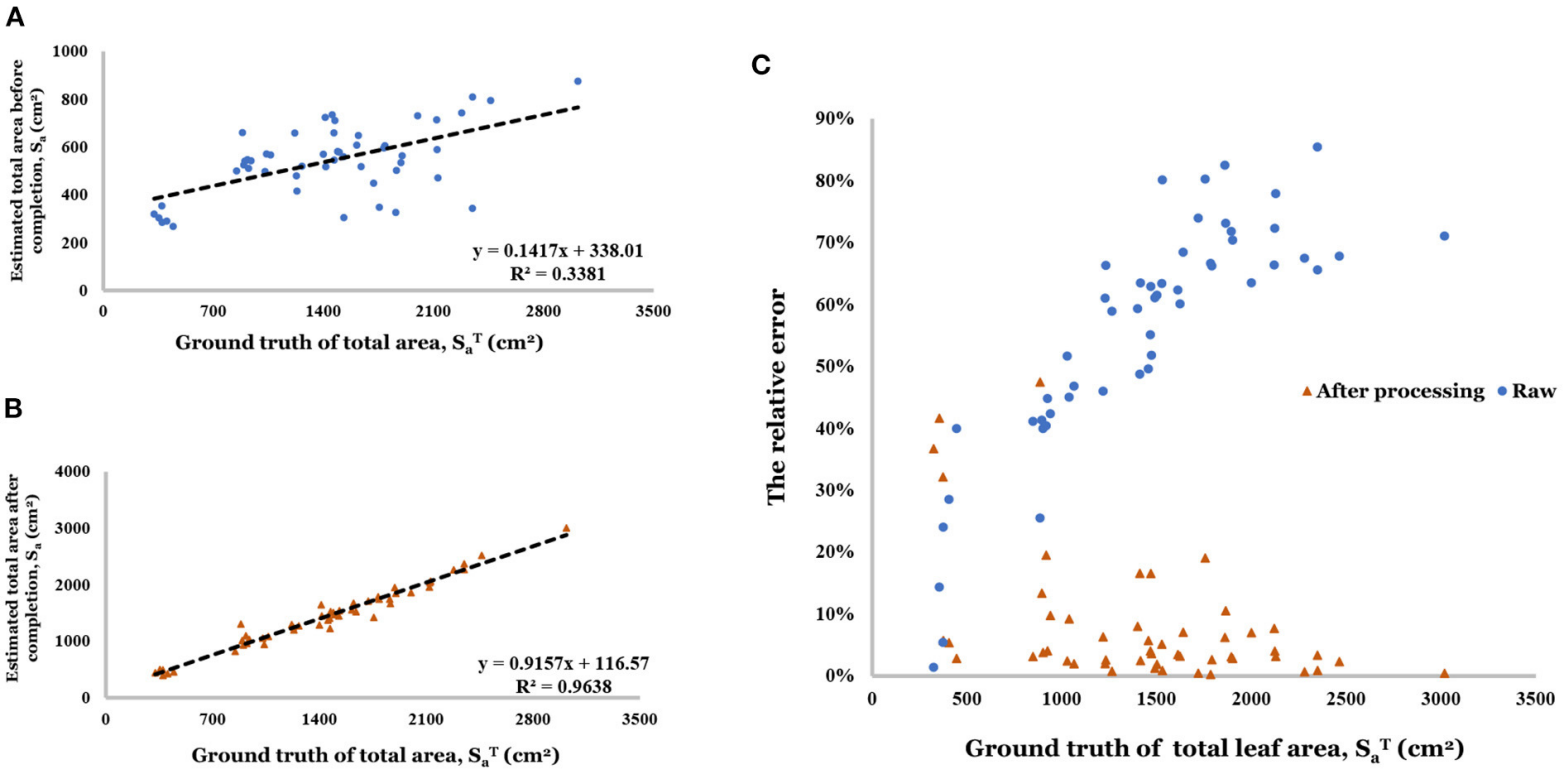
**(A)** Linear regression result of estimated total area before completion. **(B)** Linear regression result of estimated total area after completion. **(C)** Relative error of total area before and after completion.

Point cloud completion was effective for the estimation of total leaf area compared with a single view point cloud (*R*^2^ increased from 0.338 to 0.964, shown in Figures 12A,B). Especially in the range greater than 500 *cm*^2^, the estimation based on raw data has a large relative error (40.0–90.0%) in Figure 12C. And the relative error decreased to the range of 0–20.0% (80% of relative error is less than 10.0%) after the completion.

**Figure 13:**
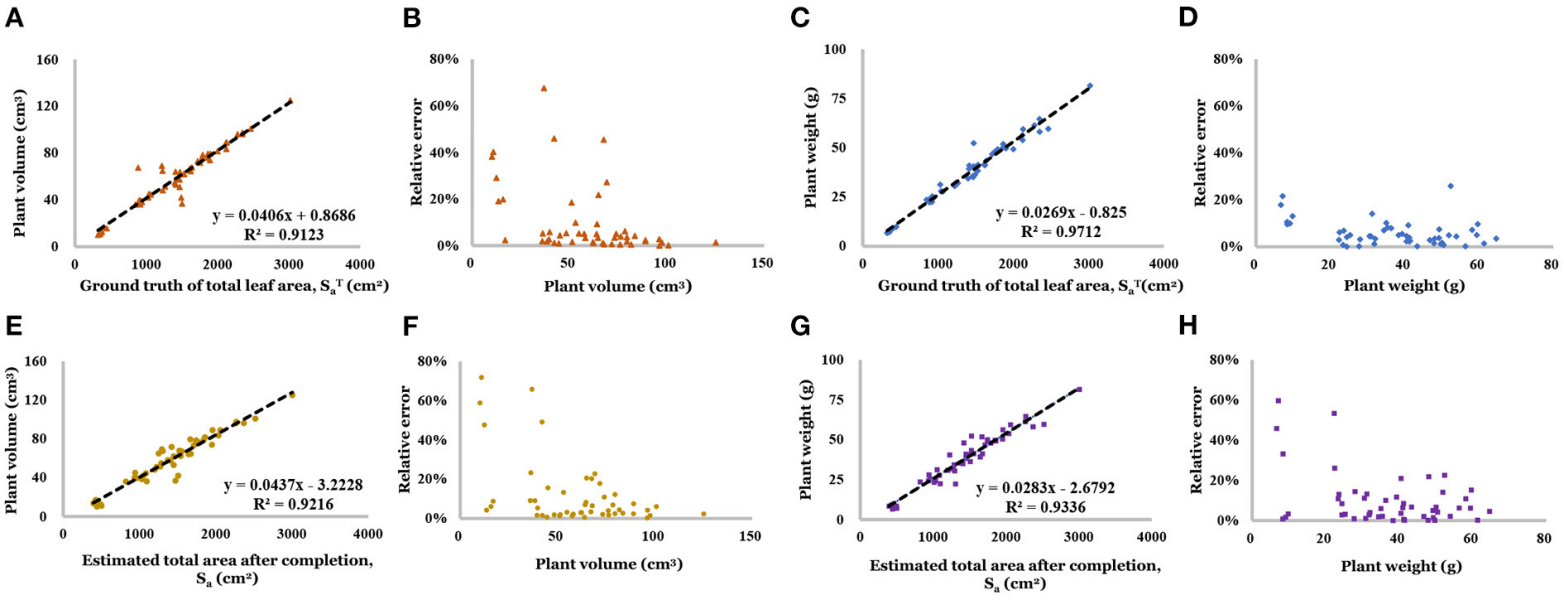
**(A)** Linear regression result of volume and ground truth of total area. **(B)** Relative error of volume (regressed by ground truth of total area). **(C)** Linear regression result of weight and ground truth of total area. **(D)** Relative error of weight (regressed by ground truth of total area). **(E)** Linear regression result of volume and estimated total area. **(F)** Relative error of volume (regressed by estimated total area). **(G)** Linear regression result of weight and estimated total area. **(H)** Relative error of weight (regressed by estimated total area).

As shown in Figures 13A,C, the ground truth leaf area is highly correlated with plant volume (*R*^2^ = 0.912) and fresh weight (*R*^2^ = 0.971), which offers the feasibility makes it a feasible tool for the weight prediction. Based on the distribution of relative error (Figures 13B,D), mast samples' predictions are close to the ground truth. And the result of the weight prediction based on the estimated total leaf area showed the high accuracy of the proposed method. Linear regression result has been showed shown in Figures 13E,G (*R*^2^ were 0.922 and 0.934, respectively). Based on the distribution of relative error (Figures 13F,H), the prediction for small size plants (less than 20 *g*) has a larger relative error (up to 70%), which means low accuracy. But 80% of the relative error concentrated on the range of 0–10.0%, which is acceptable.

## 4. Discussion

In the estimation of projection area and total leaf area, point cloud completion shows considerable effectiveness. In the estimation of projected area, *R*^2^ was raised from 0.741 to 0.911, while in the estimation of total leaf area, R2 was increased from 0.338 to 0.964. Correlating the weight with the projected area and total leaf area after point cloud completion, it was found that the total leaf area had a good linear relationship with the weight, and it was better to estimate the weight by the total leaf area. The good linear relationship between total leaf area and weight may be due to: (1) the weight of leaves per unit area is being relatively constant for this kind of leaf vegetables; (2) the total weight is being mainly concentrated on leaves.

The average point number of the plant point cloud used in the experiment was 8,336, and the processing time of point cloud was 1.048 s on average, with the longest time being 2.071 s. The average time of parameter extraction from the model was 0.579 s, and the longest time was 1.068 s. Therefore, in the whole process of data collection and extraction parameters, it will take no more than 4 s for a single plant, which can meet the demand application.

However, some problems are also found which can be addressed in the future work: (1) First, the linear correlation between true and predicted values of absolute plant height was poor. The reason is that the soil plane was not completely horizontal, and the selected soil position might affect the measured value, leading to the increase of error. (2) Also, the error between the real value and the projected area calculated after completion was very small, but it was generally slightly lower than the real value. The problem may lie in the simplification of the calculation of projected area. (3) In addition, the total leaf area obtained by point cloud calculation after completion is was still smaller than the real value, because the completion algorithm is was aimed at partially occluded leaves, which is was insufficient for more complex cases. However, there is was a good linear relationship between the total leaf area calculated after completion and the actual leaf area, and the total leaf area can still be estimated effectively through linear fitting after the linear equation is constructed in advance. Finally, (4) Our proposed method completed the 3D model of individual plant and got more accurate growth parameters. While plants grow in dense clusters in plant factoriesy, it's it is necessary to conduct segmentation first. At an early stage of the plant growth, it's it is feasible to segment an individual plant, b. Because there is space between plants and all the overlaps come from the plant itself. However, when plants clusters are too dense to segment as an individual plant, our method will encounter challenges. More effort is needed to address this problem in the future.

In this paper, we proposed a method to obtain and complete the point cloud of lettuce from a single perspective. Then we collected the data and measured the truth value of about 50 lettuces. After filtering, segmentation, and completion of the original point cloud data, the plant height, projected leaf area, and total leaf area were calculated, and linear regression was carried out with the actual values. It was found that there was a good correlation between them. In addition, we also conducted linear regression between total leaf area and actual volume and fresh weight, and found a good correlation as well, which means, after obtaining the linear equation of the corresponding plant, the plant height, leaf area, volume, and fresh weight can be estimated through the point cloud. In the future work, more considerations will be taken for small- sized plants to obtain a better performance and also the improvement of usability for the plant factory scenario.

## Data availability statement

The raw data supporting the conclusions of this article will be made available by the authors, without undue reservation.

## Author contributions

ML and JL contributed the analysis of data and design of the study. LW provided the data set and constructed environment platform. HJ supervised the research and guided the research aims. MZ guided the paper writing and revised the manuscript. All authors contributed to the article and approved the submitted version.

## Funding

This work was supported by the National Natural Science Foundation of China with Grant Nos. 31870347 and 32101626.

## Conflict of interest

The authors declare that the research was conducted in the absence of any commercial or financial relationships that could be construed as a potential conflict of interest.

## Publisher's note

All claims expressed in this article are solely those of the authors and do not necessarily represent those of their affiliated organizations, or those of the publisher, the editors and the reviewers. Any product that may be evaluated in this article, or claim that may be made by its manufacturer, is not guaranteed or endorsed by the publisher.
